# Effect of maternal eating disorders on mother‐infant quality of interaction, bonding and child temperament: A longitudinal study

**DOI:** 10.1002/erv.2960

**Published:** 2022-12-05

**Authors:** Maria Giulia Martini, Emma Taborelli, Abigail Easter, Amanda Bye, Ivan Eisler, Ulrike Schmidt, Nadia Micali

**Affiliations:** ^1^ Institute of Psychiatry, Psychology and Neuroscience (IoPPN) King's College London London UK; ^2^ Children and Young People Eating Disorder Service Central and North West London NHS Foundation Trust London UK; ^3^ Great Ormond Street Institute of Child Health University College London UK; ^4^ Maudsley Centre for Child and Adolescent Eating Disorders (MCCAED) Maudsley Hospital London UK; ^5^ South London and Maudsley NHS Foundation Trust London UK; ^6^ Department of Psychiatry University of Geneva Geneva Switzerland; ^7^ Mental Health Services in the Capital Region of Denmark Eating Disorders Research Unit Psychiatric Centre Ballerup Ballerup Denmark

**Keywords:** bonding, CARE INDEX, child temperament, eating disorder, perinatal

## Abstract

**Aims:**

This study aims to investigate the effect of maternal eating disorders (ED) on mother‐infant quality of interaction at 8 weeks and bonding and child temperament at 1 and 2 years postnatally. We also aimed to explore the relationship between maternal ED psychopathology, comorbid psychiatric difficulties, and both mother‐infant quality of interaction and bonding in women with ED. Women were recruited to a prospective longitudinal study. By the time of giving birth, the sample consisted of 101 women of the initial 137 (73.7%). Overall, 62 women (ED = 36; HC = 26) participated in the 8‐week assessment, 42 (ED = 20; HC = 22) at 1 year, and 78 (ED = 34; HC = 44) at 2 years. Mann‐Whitney *U* Test was used to explore association between maternal ED and mother‐infant quality of interaction and between maternal ED and bonding. Spearman correlations were used to explore associations between maternal ED psychopathology, comorbid psychiatric difficulties, and both mother‐infant quality of interaction and bonding.

**Results:**

We found no differences between early mother‐infant interaction and bonding in mothers with ED in comparison to HC. High levels of maternal ED psychopathology were correlated with high anxiety levels, higher negative affectivity, and lower extraversion in children of ED mothers both at 1 and 2 years. Furthermore, high levels of ED psychopathology were also associated with lower effortful control at 1 year.

**Conclusions:**

Findings imply that maternal ED have an impact on child temperament. Future research should focus on resilience and on which protective factors might lead to positive outcomes. These factors can be then used as therapeutic and preventative targets.

## Key points

- Our study did not find significant differences in each domain of mother‐infant quality of interaction and bonding between cases and controls.
- Positive correlations were found between high levels of maternal ED psychopathology, anxiety, and high levels of negative affectivity, and lower extraversion in children at both 1 and 2 years, and ED psychopathology and lower effortful control at 1 year.
- Children's temperament is compromised in the presence of a maternal eating disorder. Detection and early intervention are recommended to facilitate mother‐child interactions and to prevent any later adverse outcomes.

**Abbreviations**
AN: anorexia nervosa
BDI: Beck Depression Inventory
BED: binge eating disorder
BN: bulimia nervosa
C‐ED: Current Eating Disorder
CI: Confidence Interval
DSM‐IV: Diagnostic and Statistical Manual of Mental Disorders
ECBQ: Child temperament was measured using the Early Childhood Behaviour Questionnaire
ED: Eating Disorder
EDE‐Q: Eating Disorder Examination Questionnaire
HC: Healthy Controls
MIBS: Mother‐to‐Infant Bonding Scale
NEST‐p: The Nutrition and Stress in Pregnancy study
OR: Odds ratio
P‐ED: Past Eating Disorder
SCID‐I: Structured Clinical Interview for DSM‐ IV‐TR Axis I Disorders
SLaM: South London and Maudsley NHS Foundation Trust
STAI: The Spielberger State‐Trait Anxiety Inventory

## 1 INTRODUCTION

A maternal eating disorder (ED) is a known risk factor for feeding problems in children (Hoffman et al., 2014; Micali et al., 2011; Patel et al., 2002). A growing body of evidence suggests that children of mothers with eating disorders (ED) are also more likely to have adverse developmental outcomes, including difficult temperament, emotional and behavioural problems, and cognitive difficulties (Martini et al., 2020). Family and twin studies have consistently demonstrated that ED have a strong genetic component (Mazzeo et al., 2005) with estimates of heritability of ED ranging between 0.28 and 0.74 (Yilmaz et al., 2015). The last decade has seen an increase in research focussing on deepening our understanding of genetics and neurobiology in ED (see Bulik et al., 2022 for review). However, the environment is likely to influence the activation of genetic predispositions (Watson et al., 2018), and there is a need to examine the potential environmental mechanisms through which adverse child outcomes might develop.

In an early review of the literature on children of parents with ED carried out by Patel et al. (2002), the authors proposed that in addition to genetic influences, ED may be transmitted from parents to children as a result of both indirect effects of ED symptomatology on general parenting functioning (e.g. due to preoccupation with food, shape and weight), and direct effects on parenting (e.g. influencing the feeding choices parents make). In addition, parents may model maladaptive eating attitudes and behaviours to their children (Palfreyman et al., 2013). Previous research has shown that maternal ED may be associated with marital and family discord, which in turn have been shown to adversely influence child development (Arcelus et al., 2012). Some of the challenges women with ED might experience, seem to be directly linked to ED symptoms. For instance, mothers report avoidance of social situations due to fear of eating in public (Stitt & Reupert, 2014), concerns about their children's weight (Martini et al., 2018), and difficulties around making food choices for their children (Bryant‐Waugh et al., 2007). Mothers with ED have reported perceiving themselves incapable or inadequate in their parental role (Franzen & Gerlinghoff, 1997) and have reported having difficulties in setting boundaries (Stitt & Reupert, 2014).

A better understanding of the effects of maternal ED on child development can help identify both risk and resilience factors that could be targeted for effective therapeutic and preventative strategies, particularly identifying the pathways of risk that are modifiable.

Research in perinatal psychiatry has suggested that a modifiable pathway of risk is through parenting behaviours in early mother‐infant interactions (Stein et al., 2014). Mothers with ED report more problems in adapting to the new maternal role 3 months after delivery (Koubaa et al., 2008) and research from our group showed infants of mothers with past or current ED are exposed to higher levels of maternal anxiety, depression, and ED symptoms at 8 weeks and 6 months after birth compared to controls (Easter et al., 2015). Depressed mothers may be experienced by their children as unresponsive, inconsistent, unavailable and rejecting, displaying lower maternal sensitivity defined as the mother's ability to read the child's behavioural signals and to select and provide appropriate responses (Campbell et al., 2004). Maternal sensitivity is predictive of a wider array of child outcomes, such as better emotional and physiological regulation, lower levels of aggression, behavioural problems and a more secure mother‐infant attachment (Stein et al., 2014).

Evidence from both the Danish National Birth Cohort and the Norwegian MoBa cohort suggests that mothers with ED were more likely to perceive their infant and children as having a difficult temperament when compared to controls (Barona et al., 2016; Zerwas et al., 2012). An association might exist between maternal ED and infant negative affectivity whereby comorbid maternal depression might play an important role. Furthermore, maternal depression in mothers with ED, could also negatively bias their perception of their infant (Zerwas et al., 2012).

Only a few studies have examined mother‐child interaction in ED mothers and their infants (Sadeh‐Sharvit et al., 2016; Stein et al., 1994) but they are limited by small sample size and the majority has a cross sectional design. To the best of our knowledge, no studies have explored mother‐infant quality of interaction at a very early developmental age (birth to 6 months) and no studies have focussed on mother‐child bonding in mothers with ED.

The aim of this study was to compare (1) mother‐infant quality of interaction at 8 weeks postnatal; (2) mother‐child bonding at 1 and 2 years postnatally. (3) child temperament at 1 and 2 years postnatally amongst women with ED (both current [C‐ED] and past [P‐ED]) and Healthy Controls (HC).

Further aims were to explore the relationship between maternal ED psychopathology, comorbid psychiatric difficulties, and mother‐infant quality of interaction and to investigate the relationship between ED psychopathology, comorbid psychiatric psychopathology, and bonding in women with ED. Lastly, a further aim of the study was to investigate whether maternal C‐ED versus P‐ED would have differential effects on child outcomes. We hypothesise that mothers with histories of ED will be rated as having poorer maternal sensitivity and having more bonding difficulties with their children compared to mothers with controls. Furthermore, we hypothesise their children as being more difficult than those of mothers with no history of ED, and ED psychopathology being a predictor of such difficulties.

## 2 METHODS

### 2.1 Design and participants

The Nutrition and Stress in Pregnancy study (NEST‐p) is an observational prospective study of pregnant women recruited during pregnancy and their children up to the age of 3 years old. The overall aim was to investigate in utero mechanisms and pathways related to adverse perinatal and infant outcomes in the offspring of mothers with ED (Easter et al., 2015).

Three groups of women were recruited in this study during the first and second trimester of pregnancy: women with C‐ED, women with P‐ED and HC. The initial core NEST‐p sample consisted of 137 mothers, of whom 37 had a C‐ED, 39 had a P‐ED, and 61 were HC.

For detailed recruitment methods and study design, see (Easter et al., 2013, 2015).

Women were recruited via the following three methods:

- Women attending their first or second routine ultrasound scan at the Harris Birthright Research Centre for Fetal Medicine at King's College Hospital.
- Women referred during pregnancy to a specialist psychiatric service for treatment of an ED [that is, South London and Maudsley NHS Foundation Trust (SLaM) Perinatal Psychiatry services or SLaM ED Inpatient and Outpatient Services].
- Advertisements: Posters and leaflets were displayed in the obstetric services at KCH, perinatal psychiatry departments and SLaM ED services. Also, the information of the study was available online www.iop.kcl.ac.uk.

Participants in the C‐ED and P‐ED groups were recruited via all three methods whilst HC were recruited via methods one and three only.

For the purpose of this study, assessments were conducted in the post‐natal period at 8 weeks, 1‐ and 2‐year postnatally.

In this study, only women with complete data on predictors and outcomes at 8 weeks postpartum were included in the analyses on the impact of maternal ED on mother‐infant quality of interaction (*n* = 62 respectively: C‐ED = 16; P‐ED = 20; HC = 26), in the analyses on the impact of maternal ED on bonding at 1 year (*n* = 42, C‐ED = 7; P‐ED = 13; HC = 22) and at 2 years (*n* = 78, C‐ED = 12; P‐ED = 22; HC = 44) and child temperament at 1 year (*n* = 42, C‐ED = 7; P‐ED = 13; HC = 22; respectively) and at 2 years (*n* = 78, C‐ED = 12; P‐ED = 22; HC = 44). Within the C‐ED group, 10 (62.5%) women met criteria for anorexia nervosa (AN), 5 (31.25%) for bulimia nervosa (BN), and 1 (6.25%) for binge eating disorder (BED). Within the P‐ED group, 10 (50%) met criteria for AN, 8 (40%) for BN, and 2 (10%) for BED.

### 2.2 Inclusion criteria

Inclusion criteria for the index group were women with a past or active ED diagnosis according to the DSM‐IV, between the ages of 18 and 45 and who were within the first or second trimester of gestation.

For the HC group, the inclusion criteria were as follows: no active or past psychiatric disorder including ED, between the ages of 18 and 45, and between 12 and 25 weeks' gestation.

With the purpose of minimising the effect of confounding from active psychiatric pathology, women with psychotic illness or with another active psychiatric illness, amongst women with past ED, were excluded. In addition, women were excluded if they suffered from any chronic medical disorder or were unable to communicate in English.

### 2.3 Measures

#### 2.3.1 Predictors

##### Maternal Eating Disorder

###### ED diagnosis.

ED diagnoses were determined at baseline and were derived using the Structured Clinical Interview for DSM‐ IV‐TR Axis I Disorders (SCID‐I; American Psychiatric Association, 2000), administered by trained researchers. Interviewers were trained in using the SCID training pack. Researchers attended a monthly meeting with the senior author (N.M.), where interviews of symptomatic women were discussed. All diagnoses were reviewed and confirmed by the senior author. Interviewers demonstrated excellent interrater reliability on the SCID with 100% agreement.

###### Eating Disorder Examination Questionnaire (EDE‐Q).

At 8 weeks, 6 months, 1 and 2 years, ED symptoms were assessed using the Eating Disorder Examination Questionnaire (EDE‐Q), a 36‐item self‐report version of the EDE (Cooper & Fairburn, 1987), evaluating eating psychopathology, focussing on the last 4 weeks. In addition to a total score, the EDE‐Q measures four aspects of ED symptomatology: dietary restraint, eating concern, and body weight and body shape concerns. The EDE‐Q is a reliable and valid measure of ED psychopathology (Fairburn & Beglin, 1994).

#### 2.3.2 Outcomes

##### Mother‐baby interaction

The CARE‐Index is a developmental assessment based on the Dynamic‐Maturational Model of information processing and self‐protective organisation (Crittenden, 1996, 2002; Crittenden & Claussen, 2000b) developed by Crittenden (1981) to observe and rate the dyad's relationship. Specifically, it was devised to detect adult sensitivity to the baby in a specific dyadic context (i.e. based on the premise that the same adult could behave differently with another infant). It rates the dyad's behaviours (like facial expression, vocal expression, position and body contact, expression of affection) and cognitions (like pacing of turns, control of the activity, and developmental appropriateness of the activity).

In our study, the interactions between mother and baby were videotaped for three to 5 minutes; all mothers were invited to have a 3‐min unstructured play‐session with their baby, which was video‐recorded. They were asked to play and talk with their baby as they would normally, preferably without the use of toys. Video recordings were taken in a familiar environment, at the participant's house. All videos were later scored by a researcher, who was blind to participant status (i.e. the researcher was not aware if the video they were scoring depicted ED cases or HC) and trained in scoring the CARE‐Index recordings. Another blinded researcher was asked to score all the videos to measure inter‐rater reliability. The interclass correlation coefficient showed high inter‐reliability between the two raters for both maternal and infant subscales, ranging between 0.90 and 0.97. The behaviours of both mother and baby were coded on seven 14‐point scales, three for the mother: Adult Sensitivity, Control, and Withdrawal and four for the infant: cooperation, difficultness, passivity, and compulsive compliance (Crittenden, 1981, 1985). The Care index scales were not normally distributed and could not be normalised after transformation.

##### Maternal to infant bonding scale

The Mother‐to‐Infant Bonding Scale (MIBS) (Taylor et al., 2005) was designed as a self‐report questionnaire. It was given to participants asking them about their feeling towards their baby in the first few weeks after they were born. It consists of eight statements describing an emotional response, such as ‘‘loving’’ or ‘‘disappointed’’ rated on a 4‐point scale from very much (score = 0) to not at all (score = 3). Scores can range from 0 to 24. Five items describe negative emotional responses (i.e. resentful, neutral or felt nothing, dislike, disappointed and aggressive) and are reverse scored. A total score was derived by summing each item, with low scores denoting good bonding and high scores indicating problems in the mother‐infant bonding. MIBS total score was not normally distributed and could not be normalised. The MIBS can be used in the first weeks after the child's birth to identify difficulties experienced by the mother in establishing a relationship with the baby. The MIBS has an acceptable internal consistency (*α* = 0.71) and reasonable validity (Taylor et al., 2005).

##### Child temperament

Child temperament was measured using the Early Childhood Behaviour Questionnaire (ECBQ) a self‐administrated questionnaire. The ECBQ was designed as an adapted form of the Toddler Behaviour Assessment Questionnaire (TBAQ, Goldsmith, 1996) used by parents to report their infant's temperament in terms of individual differences in emotionality during early development. In addition, the ECBQ includes scales developed by Mary Klevjord Rothbart contain other situations involving, besides emotions, motor and sensory systems and self‐regulatory practices that control reactivity (Putnam et al., 2006; Rothbart et al., 2001). It has been validated by Putman and colleagues.

The ECBQ has 201 items that measure 18 scales; activity level, attention shifting and focussing capability, cuddliness, discomfort, fear, frustration, high and low intensity pleasure, impulsivity, inhibitory control, perceptual sensitivity, positive anticipation, sadness, soothability, shyness, motor activation and sociability. The questionnaire contains three main factors of temperament: (1) Negative affectivity, which is a broad personality trait that involve the experience of negative emotions (i.e. fear, sadness etc) and poor self‐concept; (2) Surgency/Extraversion which is a trait aspect of emotional reactivity where a person tends to experience high levels of positive affect (i.e. cheerfulness, sociability etc). Due to the word “Surgency” being unfamiliar, “Extroversion” will be used throughout the paper; 3. Effortful Control which is the child's ability to use attentional resources and to inhibit behavioural responses in order to regulate emotions and related behaviours. Answers are rated from never, very rarely, less than half the time, about half the time, more than half the time, almost always and always. Each answer was scored from 1 to 7. The scores for items receiving a numerical response were summed. For participants with less than 25% missing data, total scores were calculated using prorating (Strube, 1985). ECBQ subscales were normally distributed and used as continuous variables.

#### 2.3.3 Covariates

##### Socio‐demographic data

Maternal age, maternal education, ethnicity, marital status, and parity were obtained via self‐report at enrolment into the study. Parity was dichotomised into nulliparous versus primiparous/multiparous. Marital status was coded as a binary variable into single with or without a partner versus married/cohabiting. Ethnicity was dichotomised into Caucasian versus non‐Caucasian. Maternal education was coded as A level^1^ or higher (secondary school or above), and lower than A level (no secondary school qualification).

##### Depressive symptoms

The Beck Depression Inventory (BDI; Beck et al., 1961) was used to assess women's symptoms of depression at 12, 25–28 weeks, 32 weeks antenatally and 8 weeks, 6 months, 1 and 2 years postnatally. The BDI is a 21‐item multiple choice self‐reported measure of the intensity and severity of depression. Questions relate to how the respondent has felt within the previous week. A composite variable was derived on the basis of individuals scoring having any clinical depression (total score>20) at any of the above point.

##### Anxiety

The Spielberger State‐Trait Anxiety Inventory (STAI) is a frequently used measure for self‐reported anxiety (Spielberger et al., 1983). The STAI consists of two separate 20‐item scales of anxiety: state anxiety (which is considered to be relatively temporary and transient) and trait anxiety (which is considered to be more long‐standing and stable). It was used at 12, 25–28 and 32 weeks antenatally, and 8 weeks, 6 months, 1 and 2 years postnatally. A composite variable was calculated if women scored as having clinical anxiety (total score>38) at any point.

##### Ethical approval

The Nest‐p project received NHS ethical approval by The Joint South London and Maudsley and the Institute of Psychiatry Research Ethics Committee (Ref. 09/H0807/12).

### 2.4 Statistical analysis

#### 2.4.1 Primary analyses

Outcomes and covariates were investigated in terms of distribution.

Mann‐Whitney *U* Test was used to explore association between maternal ED and mother‐infant quality of interaction and between maternal ED and bonding.

C‐ED and P‐ED were grouped together given previous evidence that the effects of current or past ED on child development are similar (Barona et al., 2017; Hoffman et al., 2014).

Crude and adjusted linear regression analyses were used to test whether maternal ED were prospectively associated with child temperament. A partially adjusted model included the following: maternal age, ethnicity, education, marital status, parity, as a priori confounders. BDI and STAI were subsequently included in the fully adjusted model to investigate their mediating role on the association between maternal ED and child temperament.

Crude and adjusted linear regression analyses were run to explore whether ED psychopathology at 6 months postnatally was predictive of child temperament at 1 and 2 years postnatally. A partially adjusted model included the following: maternal age, ethnicity, education, marital status, parity, as a priori confounders. BDI and STAI were subsequently included in the fully adjusted model to investigate their mediating role on the association between ED psychopathology and child temperament.

Spearman correlations were run, in the ED group only, between maternal sensitivity, bonding and ED psychopathology, depressive and anxiety symptoms at 6 months postnatally to explore the cross‐sectional effect of maternal ED symptoms, comorbid psychiatric difficulties and mother‐infant quality of interaction and bonding.

Likewise, Spearman correlations were run, in the ED group only, between child temperament and ED psychopathology at 6 months postnatally, depressive and anxiety symptoms to investigate cross‐sectional associations of maternal ED symptoms, comorbid psychiatric difficulties and child temperament.

#### 2.4.2 Post‐hoc analyses

Further post hoc analyses were conducted in order to investigate associations between maternal C‐ED and P‐ED versus HC and outcomes under study. However, due to the small sample size within these subgroups (particularly C‐ED subgroup, *N* = 7), these comparisons should be considered largely exploratory. Furthermore, to account for multiple comparisons, *p*‐values of analyses within these subgroups' post hoc tests were adjusted using the Bonferroni correction method.

Kruskal Wallis one‐way ANOVA was used to explore association between maternal ED (C‐ED and P‐ED vs. HC) and mother‐infant quality of interaction.

Spearman correlations were run, in the C‐ED and P‐ED group separately, between bonding and ED psychopathology, depressive and anxiety symptoms at 6 months postnatally to explore the cross‐sectional effect of maternal ED symptoms, comorbid psychiatric difficulties and bonding.

Spearman correlations were run, in the C‐ED and P‐ED group separately, between infant temperament and ED psychopathology, depressive and anxiety symptoms at 6 months postnatally to explore the cross‐sectional effect of maternal ED symptoms, comorbid psychiatric difficulties and bonding.

All analyses were performed using SPSS 26 (IBM Corp, 2019) and a two‐tailed significance level of *p* ≤ 0.05 was used.

### 2.5 Attrition and missingness

By the time of giving birth, the sample consisted of 101 women of the initial 137 (73.7%). Overall, 62 women (62.6% of total) participated in the 8‐week assessment, at 1 year, 42 (42.4%) and 78 (78.8%) at 2 years. Attrition is to be expected in longitudinal studies. We investigated differences between women with complete data and those with missing data on outcomes; missingness at each time point was not predicted by ED status, maternal age, education or marital status. Missing data on outcomes were, therefore, assumed to be at random.

We investigated missingness among women with complete data on predictor and outcome at each time point separately. Multiple random imputation was used to deal with missing confounder data, and both predictors and outcomes were used in the model. Missing data were imputed for maternal education, ethnicity, marital status, and parity.

Results for both complete and imputed cases were almost identical; consequently, the results based on the imputed cases are presented here as multiple imputation is assumed to correct bias.

## 3 RESULTS

### 3.1 Socio‐demographics characteristics

The majority of women in the sample were from a white ethnic background (87.1%), were married or cohabiting at the time of recruitment (82.3%), and had a university degree or higher education (90.9%). No differences in the ethnicity, education, relationship status or parity between women with ED and HC were found (see Table 1). Maternal age significantly varied across the three groups, the C‐ED group were significantly younger than P‐ED and HC in post‐hoc analyses (*F* = 5.16; *p* = 0.009).

**TABLE 1:** Maternal demographic variables in women with ED versus healthy controls

	ED	HC	Statistic, *p*‐value
	*N* = 36	*N* = 26	
Ethnicity %			
White	30 (83.3%)	24 (92.3%)	*p* = 0.48
Other ethnic background	6 (16.7%)	2 (7.7%)	
Marital status %			
Single	9 (25%)	2 (7.7%)	*p* = 0.15
Married/cohabitating	27 (75%)	24 (92.3%)	
Education %			
No formal education/GCSEs	4 (12.9%)	1 (4.2%)	*p* = 0.43
A levels/higher	27 (87.1%)	23 (95.8%)	
Maternal age, mean (SD)	31.92 (5.70)	33.92 (4.04)	3.32, *p* = 0.16

*Note*: Fisher tests were undertaken for categorical outcomes and continuous outcomes were tested using analysis of variance.

*p < 0.05; **p < 0.01; ***p < 0.001.

### 3.2 Mother‐infant quality of relationship at 8 weeks old

No significant differences were found in each domain of mother‐child quality of interaction between cases and controls both in primary (ED grouped together) (see Table S1) and post‐hoc analyses (C‐ED and P‐ED vs. HC). Spearman correlations did not reveal any significant associations between maternal sensitivity and ED psychopathology, depressive, and anxiety symptoms (see Table S3).

### 3.3 Mother‐infant bonding at 1 and 2 years postnatally

ED women had higher MIBS scores (worse bonding) than controls both at 1 and 2 years postnatally, although this was not statistically significant (see Table S2).

Maternal ED was not predictive of poorer bonding at 1 and 2 years postnatally and results remained non‐significant when adjusting for depression and anxiety scores. However, Spearman correlations showed positive correlations between MIBS score and depressive symptoms (Rs = 0.43, *p* = 0.04) at 1 year. At 2 years, a positive correlation was observed both between maternal anxiety state symptoms, and depressive symptoms and MIBS score (Rs = 0.36, *p* = 0.04; Rs = 0.31, *p* = 0.02 respectively) (see Table 2). Maternal shape concerns predicted worse bonding at 1 year in the crude regression model (OR = 1.57, 95% CI (1.01–2.45), *p* = 0.04) but the difference was no longer significant in partially and fully adjusted models. No other significant differences were found in either the partially and fully adjusted models. In our post‐hoc analyses, positive correlations were observed between MIBS within the P‐ED subgroup and maternal weight concerns both at 1 and 2 years (Rs = 0.56, *p* = 0.01 and Rs = 0.57, *p* = 0.01 respectively). Positive correlations were also found in the C‐ED subgroup between MIBS and depressive symptoms at 1 year (Rs = 0.81, 95% CI (0.18–0.97)) and anxiety levels at both 1 at 2 years (Rs = 0.84, 95% CI (0.24–0.98); Rs = 0.84, 95% CI (0.27–0.98) respectively).

**TABLE 2:** Spearman correlations between bonding and ED psychopathology (EDE‐Q), depressive symptoms (BDI), anxiety symptoms (STAI) at 1 and 2 years in women with ED

	*N* Tot	Maternal‐infant bonding at 1 year	*N* Tot	Maternal‐infant bonding at 2 years
Restrain subscale	20	Rs = 0.16	34	Rs = 0.13
Eating concern subscale	20	Rs = 0.44	34	Rs = 0.39
Weight concern subscale	20	Rs = 0.26	34	Rs = 0.26
Shape concern subscale	20	Rs = 0.42	34	Rs = 0.42
State Anxiety (STAI)	20	Rs = 0.38	34	Rs = 0.36*
Trait Anxiety (STAI)	20	Rs = 0.18	34	Rs = 0.29
Depressive symptoms (BDI)	20	Rs = 0.43*	34	Rs = 0.31*

*p ≤ 0.05; **p ≤ 0.01; ***p ≤ 0.001.

### 3.4 Impact of maternal ED on child temperament at 1 and 2 years postnatally

Maternal ED was not predictive of child temperament in the domains of negative affectivity, surgency, and effortful control at 1 and 2 years (see Table 3).

**TABLE 3:** Effect of maternal ED on child behaviour in the domain of negative affectivity, extraversion, effortful control at 1 and 2 years: linear regression, coefficient (B), and 95% confidence intervals (CI)

	Crude (B, 95%)			Partially adjusted^a^ (B, 95%)			Fully adjusted^b^ (B, 95%)		
	Year 1								
ED status	Negative affectivity	Extraversion	Effortful control	Negative affectivity	Extraversion	Effortful control	Negative affectivity	Extraversion	Effortful control
HC *N* = 22	Ref.	Ref.	Ref.	Ref.	Ref.	Ref.	Ref.	Ref.	Ref.
ED, *N* = 20	0.15 (−0.14 to 0.43)	0.11 (−0.19 to 0.40)	0.01 (−0.30 to 0.32)	0.16 (−0.13 to 0.46)	0.07 (−0.28 to 0.41)	0.21 (−0.11 to 0.53)	0.15 (−0.32 to 0.61)	0.17 (−0.28 to 0.63)	0.32 (−0.20 to 0.84)

	Crude (B, 95%)			Partially adjusted^a^ (B, 95%)			Fully adjusted^b^ (B, 95%)		
	Year 2								
HC *N* = 44	Ref.	Ref.	Ref.	Ref.	Ref.	Ref.	Ref.	Ref.	Ref.
ED, *N* = 34	0.19 (−0.28 to 0.52)	0.23 (−0.08 to 0.54)	−0.45 (−0.83 to 0.08)	0.17 (−0.20 to 0.54)	0.20 (−0.12 to 0.53)	−0.47 (−0.86 to 0.04)	0.45 (−0.04 to 0.93)	0.21 (−0.19 to 0.49)	−0.45 (−1.09 to 0.20)

^a^ Model 2: adjusted for age, ethnicity, education, marital status and parity.

^b^ Model 3: adjusted for age, ethnicity, education, marital status, parity, BDI, STAI.

*p ≤ 0.05; **p ≤ 0.01; ***p ≤ 0.001.

Correlation analyses revealed positive correlations between negative affectivity and higher ED psychopathology scores, whilst negative correlations were observed between shape concerns and both extraversion and effortful control at 1 year (see Table 4). The negative affectivity score was positively correlated with the eating restraint subscale and negative correlated with the extraversion subscale (see Table 4).

**TABLE 4:** Spearman correlation between infant temperament at 1 and 2 years and ED psychopathology (EDE‐Q) at 6 months postnatally, depressive symptoms (BDI), anxiety symptoms (STAI) in ED group

		Negative affectivity	Extraversion	Effortful control		Negative affectivity	Extraversion	Effortful control
Predictor	N Tot	Year 1			N Tot	Year 2		
Restrain subscale	20	Rs = 0.72**	Rs = −0.26	Rs = −0.16	20	Rs = 0.59*	Rs = −0.53	Rs = −0.62*
Eating concern subscale	20	Rs = 0.80**	Rs = −0.51	Rs = −0.20	20	Rs = 0.21	Rs = −0.51	Rs = 0.50
Weight concern subscale	20	Rs = 0.66**	Rs = −0.42	Rs = −0.29	20	Rs = 0.14	Rs = 0.48	Rs = 0.47
Shape concern subscale	20	Rs = 0.72**	Rs = −0.53*	Rs = −0.27*	20	Rs = 0.21	Rs = 0.48	Rs = 0.44
Composite STAI‐STATE	20	Rs = 0.44	Rs = −0.02	Rs = −0.26	20	Rs = −0.42	Rs = −0.25	Rs = 0.27
Composite STAI‐TRAIT	20	Rs = 0.17	Rs = −0.21	Rs = −0.02	20	Rs = −0.14	Rs = 0.34	Rs = 0.16
Composite BDI	20	Rs = 0.56	Rs = −0.29	Rs = −0.39	20	Rs = −0.46	Rs = 0.20	Rs = 0.31

*p ≤ 0.05; **p ≤ 0.01; ***p ≤ 0.001.

Maternal ED psychopathology predicted higher negative affectivity in children at 1 year (refer to Table 5). At 2 years, the maternal restraint subscale was predictive of higher negative affectivity in children in all three models (*b* coeff = 0.27 95% CI (0.09–0.45), *p* = 0.007), whilst weight and shape concerns predicted higher negative affectivity only in the fully adjusted models (*b* coeff = 0.34 95% CI (0.08–0.60), *p* = 0.02; *b* coeff = 0.39 95% CI (0.16–0.61), *p* = 0.003 respectively). Maternal eating concerns and maternal weight concerns were predictive of lower extraversion in the partially adjusted model at 1 year (*b* coeff = −0.35 95% CI (−0.66 to −0.32), *p* = 0.03; *b* coeff = −0.28 95% CI (−0.49 to −0.07), *p* = 0.01, respectively). Maternal shape concerns were predictive of lower extraversion in children both in the crude and partially adjusted model (*b* coeff = −0.26 95% CI (−0.46 to −0.06), *p* = 0.02). At 2 years, maternal eating concerns were predictive of lower extraversion in their children in the partially adjusted model (*b* coeff = −0.44 (−0.34–0.03), *p* = 0.03) and maternal shape concerns were predictive of lower extraversion in children both in the crude and partially adjusted models (*b* coeff = −0.44 95% CI (−0.25–0.02), *p* = 0.02). Maternal shape concerns were predictive of lower effortful control in their children at 1 year in the crude model (*b* coeff = −0.19 (−0.36 to −0.02), *p* = 0.04). No significant differences were found at 2 years.

**TABLE 5:** Maternal predictors in ED group for negative affectivity, extraversion and effortful control at 1 and 2 years: linear regression, coefficient (B), and 95% confidence intervals (CI)

	Crude (B, 95%)			Partially adjusted^a^ (B, 95%)			Fully adjusted^b^ (B, 95%)		
	Year 1, *N* = 20								
	Negative affectivity	Extraversion	Effortful control	Negative affectivity	Extraversion	Effortful control	Negative affectivity	Extraversion	Effortful control
EDE‐Q restrain	0.34 (0.15–0.53)*	−0.02 (−0.2 to 0.2)	−0.12 (−0.32 to 0.08)	0.31 (0.08–0.54)**	−0.04 (−0.29 to 0.21)	−0.05 (−0.28 to 0.19)	0.34 (0.03–0.64)*	0.003 (−0.27 to 0.28)	−0.06 (−0.35 to 0.24)
Eating concerns	0.40 (0.18–0.61)***	−0.13 (−0.34 to 0.09)	−0.19 (−0.41 to 0.03)	0.38 (0.01–0.76)*	−0.35 (−0.66 to −0.32)*	−0.14 (−0.42 to 0.28)	0.44 (−0.09 to 0.96)	−0.23 (−0.63 to 0.17)	−0.14 (−0.60 to 0.32)
Weight concerns	0.32 (0.11–0.52)**	−0.16 (−0.33 to 0.23)	−0.18 (−0.37 to 0.01)	0.29 (0.03–0.55)*	−0.28 (−0.49 to −0.07)*	−0.15 (−0.39 to −0.09)	0.43 (0.00–0.85)*	−0.26 (−0.58 to 0.06)	−0.25 (−0.61 to 0.11)
Shape concerns	0.29 (0.09–0.49)**	−0.17 (−0.33 to −0.04)*	−0.19 (−0.36 to −0.02)*	0.23 (−0.04‐ 0.49)	−0.26 (−0.46 to −0.06)*	−0.11 (−0.34 to 0.12)	0.34 (−0.13 to 0.80)	−0.19 (−0.54 to 0.15)	−0.28 (−0.63 to 0.08)

	Crude (B, 95%)			Partially adjusted^a^ (B, 95%)			Fully adjusted^b^ (B, 95%)		
	Year 2, N = 34								
EDE‐Q restrain	0.18 (0.03–0.33)*	0.02 (−0.15 to 0.16)	0.16 (−0.02 to 0.34)	0.20 (0.02–0.03)*	0.03 (−0.15 to 0.17)	0.10 (−0.12 to 0.31)	0.27 (0.09–0.45)**	0.14 (−0.02 to 0.30)	0.11 (−0.18 to 0.41)
Eating concerns	0.13 (−0.06 to 0.32)	−0.10 (−0.22 to 0.10)	0.12 (−0.10 to 0.34)	0.13 (−0.19 to 0.44)	−0.44 (−0.34–0.03)*	−0.07 (−0.40 to 0.27)	0.28 (−0.08 to 0.64)	0.23 (−0.02 to 0.48)	−0.17 (−0.64 to 0.29)
Weight concerns	0.08 (−0.09 to 0.25)	−0.31 (−0.21 to 0.02)	0.14 (−0.05 to 0.32)	0.05 (−0.18 to 0.28)	−0.31 (−0.22 to 0.03)	0.04 (−0.19 to 0.28)	0.34 (0.08–0.60)*	0.08 (−0.10 to 0.27)	0.02 (−0.39 to 0.43)
Shape concerns	0.10 (−0.06 to 0.26)	−0.43 (−0.24 to 0.02)*	0.14 (−0.04 to 0.31)	0.08 (−0.13 to 0.29)	−0.44 (−0.25 to 0.02)*	0.10 (−0.12 to 0.32)	0.39 (0.16–0.61)**	0.05 (−0.13 to 0.24)	0.13 (−0.28 to 0.53)

^a^ Model 2: adjusted for age, ethnicity, education, marital status and parity.

^b^ Model 3: adjusted for age, ethnicity, education, marital status, parity, BDI, STAI.

*p ≤ 0.05; **p ≤ 0.01; ***p ≤ 0.001.

In our post‐doc analyses, in the C‐ED subgroup higher levels of maternal ED psychopathology were positively correlated with higher negative affectivity in children at 1 year (see Table S4). In the P‐ED subgroup there was a positive correlation between Negative Affectivity and both higher levels of maternal ED psychopathology and higher maternal anxiety state scores at 1 year (see Table S5). At 2 years, higher restraint was positively associated with both higher negative affectivity (*R* = 0.58, *p* = 0.02) and higher effortful control (Rs = 0.62, *p* = 0.01) (see Table S5).

## 4 DISCUSSION

To our knowledge, this is the first study that explored the relationships between maternal ED psychopathology, psychiatric comorbidities, and early mother‐infant quality of interaction in non‐feeding time at 8 weeks, and the longitudinal effects of the maternal ED on bonding and child temperament at 1 and 2 years postnatally. We found no differences between early mother‐infant interaction and bonding in mothers with ED in comparison to HC. High levels of maternal ED psychopathology were correlated with high anxiety levels, higher negative affectivity, and lower extraversion in children of ED mothers both at 1 and 2 years. Furthermore, high levels of ED psychopathology were also associated with lower effortful control at 1 year.

### 4.1 ED and mother‐infant quality of relationship

We did not find any significant differences between early mother‐infant interaction in mothers with ED compared to controls. Specifically, our findings suggest that maternal ED does not seem to have an impact in the relationship with the infant at 8 weeks aside of feeding time and that mothers with ED are able to respond to their children's cues and exhibit sensitive parenting behaviours at this stage.

In line with our results, previous research likewise showed no differences in behavioural responsiveness to children (aged 6–36 months) in mothers with histories of ED compared to controls during feeding or free‐play (Hoffman et al., 2014). However, these mothers did report more parenting stress, increased anxiety, and blunted physiological stress response, making them more vulnerable for psychological stress‐related problems when they faced additional challenges (Hoffman et al., 2014). In our study, mother‐infant dyads were assessed at 8 weeks postnatally which is a time where infants still fully rely on either breastmilk or formula milk and have not been weaned yet. One possible explanation for this finding could be that when weaning occurs, and infants become more accustomed to solid foods, mothers with ED might become less sensitive, more controlling and more hostile during play (Sadeh‐Sharvit et al., 2016) and mealtimes (Stein et al., 1994). However, in our study we were not able to assess the infant‐mother quality of relationship longitudinally and this might be something for future research to focus on.

### 4.2 ED and mother‐child bonding

We did not find maternal ED to be predictive of poorer bonding at 1 and 2 years postnatally even after adjusting for depression and anxiety. Based on previous research (Barona et al., 2016; Patel et al., 2002) we expected mothers with ED to experience more problems with mother‐to‐infant bonding than unexposed mothers. Results from this study did not support our initial hypothesis. Whilst this suggests that mothers with ED do not differ from HC in their ability to bond with their children at 1 and 2 years, our results could also be due to small sample size, leading to type II error.

However, in the index group we found positive correlations between bonding score, maternal eating concerns and depressive symptoms at 1 year and bonding score, anxiety symptoms and depressive symptoms at 2 years. In a review of the literature on ED in the post‐natal period, authors concluded that post‐natal depression in mothers with ED, intensified by residual pregnancy bodily changes, can negatively impact on mother and infant bonding and attachment in the post‐partum period (Astrachan‐Fletcher et al., 2008). Previous studies have illustrated that mothers with ED are more prone to have comorbid anxiety and depression compared to healthy controls (Easter et al., 2015; Johansen et al., 2020) and demonstrated the impact of maternal depression on mother‐infant bonding (Slomian et al., 2019). There is also evidence that depressed mothers might show less closeness, warmth, and lower level of emotional availability with their child throughout the first year postpartum (Lilja et al., 2012). The effect of a lack of “tuning in” in the interaction, in this sensitive period, can have important consequences on child cognitive and social‐emotional development and also interfere with the process of learning (Slomian et al., 2019).

Fewer studies focused on the impact of maternal anxiety on bonding with anxious mothers reporting significantly lower bonding to their infants compared to unexposed mothers (Tietz et al., 2014). In an experimental study, where cases and control mothers were randomly assigned to either a worry/rumination prime or a neutral prime, Stein et al., demonstrated that disturbances in maternal cognition in the context of anxiety (and to a lesser extent depression) play a potential causal role in the negative effects of mother‐child interaction, with their children exhibiting lower emotional tone and being more withdrawn (Stein et al., 2012).

Despite needing replication, our results did not suggest that ED symptoms have an independent and specific effect on mother‐child interaction but that comorbid depression and anxiety can impact on child bonding, which might have detrimental consequences for later child emotional and cognitive development, and could be an important mediator for transmission of ED risk. Lastly, given the high prevalence of anxiety and depression in general practice patients and the most common comorbidities in other mental health disorders (Kalin, 2020), there is scope for future research to include mothers with depression and anxiety symptoms to explore disorder‐specific child outcomes.

### 4.3 ED and infant temperament

Our study found positive correlations between high levels of maternal ED psychopathology, anxiety, and high levels of negative affectivity, and lower extraversion in children at both 1 and 2 years, and ED psychopathology and lower effortful control at 1 year. Although the literature on maternal ED and child temperament is scant, the results observed in this study replicate past research. In a large population‐based study, Barona et al., found that women with ED tended to report their children as having a more difficult temperament, characterised by their child being less happy and active than other children of their age, more restless and having more tantrums, being less cautious and more guarded than controls (Barona et al., 2016). However, because the present study used maternal report rather than direct observational measures for mother–child relation and child temperament at 1 and 2 years, the nature of the associations remains unclear. One possible explanation could be that preoccupations related to food, shape and weight can take up a considerable part of the day in mothers with high levels of ED psychopathology and can impact on the mothers' availability for their children which in turn are more likely to express feeling of sadness, fear and higher levels of frustration. Yet we cannot reject the possibility that mothers with ED may perceive their child as being more difficult and previous studies showed that mothers experiencing psychological distress have cognitive styles that may bias assessment in a negative way (i.e., black‐and‐white thinking, overgeneralisation, clinical perfectionism) (Limburg et al., 2017). However, even if these difficulties are a subjective maternal experience, perception does possibly impact on one's own sense of being a mother (Crockenberg & Acredolo, 1983) and is likely to negatively affect the child's later development.

### 4.4 Strengths and limitations

This study has many strengths. First, it is the first study to explore longitudinally the impact of maternal ED on mother‐infant relationship, bonding and infant temperament.

Our outcomes were all assessed prospectively, avoiding the recall bias that retrospective research suffers from. Furthermore, a second strength of this study is the use of the unique tool of CARE‐INDEX, that involves assessment of patterns of mother‐infant interaction through video observation rather than questionnaires. Another strength is that ED diagnoses were based on diagnostic interviews, rather than self‐report. Given our recruitment approach, women in the sample included both clinical and community cases. Therefore, the generalisability of our results might be greater than studies using samples from specialist services only and community cases only and might include women with a range of ED severity.

However, some limitations should be noted. Women who continued to take part in our study for the full two post‐natal years were more likely to be Caucasian and better educated, limiting the generalisability of results. However, no selective attrition was noted in women with ED. Another limitation lies in the difficulty in studying the effect of specific diagnoses (AN, BN, and BED) due to small sample sizes.

The overall sample size of index groups was small and could have resulted in a type II error, especially in post‐hoc testing, due to low power. Therefore, the above results should be considered preliminary and should support further studies to uncover potential differences in mother‐infant relationship, bonding, infant temperament between C‐ED and P‐ED. Lastly, despite grouping C‐ED and P‐ED together is justified by previous evidence, this could have led to a Type I error.

### 4.5 Clinical implications and conclusions

In conclusion, maternal ED did not have an impact on mother‐infant quality of relationship at 8 weeks. However, as previously discussed, these difficulties may arise later on, possibly when weaning occurs. Further research should focus on longitudinal assessment through observation‐based measures to assess the impact of maternal ED on mother‐infant relationship throughout the first years of life.

Comorbid anxiety and depression in mothers with ED were associated with worse mother‐child bonding. Given the detrimental consequences of poor mother‐child bonding on child cognitive, social emotional development and also interfering with the process of learning, we cannot stress enough the importance of close monitoring and consistency of care during pregnancy and the postpartum period. This will increase the chances to detect and treat early any psychiatric comorbidities.

Findings from our study also suggest that children's temperament is compromised in the presence of a maternal eating disorder. Children with difficult temperament have increased risk for future peer difficulties, mental health problems, more impaired social outcomes as they get older and more contacts with psychiatric services (Watson et al., 2018). Detection and early intervention are recommended to facilitate mother‐child interactions and to prevent any later adverse outcomes. Prosocial behaviours and social emotional functioning could be targeted early on through family‐centred and integrated family‐ and school‐based interventions (Johnson et al., 2014).

However future research should focus on resilience (i.e. father involvement, family support and resources, wider support network etc) and on which factors can protect the child and are amenable to be therapeutic and preventative targets.

## CONFLICT OF INTEREST

The authors declare that they have no competing interests.

## Supporting information

Supporting Information S1

## Data Availability

The data that support the findings of this study are available from the corresponding author upon reasonable request.

## ENDNOTE

^1^
In the British education system, A‐levels are subject‐based qualifications that can lead to university, further study, training, or work. These are essential prerequisite for those willing to go to University i.
