# Supplementary material for: Effect of maternal eating disorders on mother‐infant quality of interaction, bonding and child temperament: A longitudinal study
Source: Eur Eat Disord Rev. 2022 Dec 5;31(2):335–48. doi: 10.1002/erv.2960 (PMC10107506; doi:10.1002/erv.2960)
Supplement: Supplementary file 1 — Supporting Information S1 [file ERV-31-335-s001.docx]

**Table S1. Median and Interquartile range (IQR) for mother child relation (CARE Index) by groups (ED vs HC) using Mann-Whitney U Test**

|  | ED  N=36 | HC  N=26 | P |
| --- | --- | --- | --- |
| Mother sensitive | 11.00 (7.25-12.00) | 10.00 (7.00-12.00) | 0.84 |
| Mother controlling | 2.50 (1.25-4.00) | 2.50(1.00-5.00) | 0.98 |
| Mother unresponsive | 0.00 (0.00-2.75) | 0.00 (0.00-3.00) | 0.39 |
| Infant cooperative  Infant compulsive | 10.00 (6.00-13.00)  0.00(0.00-0.00) | 8.00 (6.75-12.25)  0.00 (0.00-0.00) | 0.51  0.50 |
| Infant difficult  Infant passive | 2.00(1.00-4.00)  0.00 (0.00-3.00) | 0.00 (0.00-3.50)  2.00 (0.00-6.00) | 0.07  0.12 |

*Note:* Mann-Whitney U Test was used to test median and Interquartile range (IQR) for mother child relation (CARE Index) by groups

*p≤0.05, **p≤0.01, ***p≤0.001

**Table S2. Infant bonding and infant behaviour scores at 1 and 2 years, comparisons between women with ED and HC**

| **Mean/Median scores (IQR) at 1 year** | | | **Mean/Median scores (IQR) at 2 year** | |
| --- | --- | --- | --- | --- |
|  | **ED**  **N=20** | **HC**  **N=22** | **ED**  **N=34** | **HC**  **N=44** |
| Maternal infant bonding (median, range) | 2.00 (0.25-4.00) | 0.50 (0.00-3.75) | 1.50 (0.00-4.00) | 0.00 (0.00-2.00) |
| Infant behaviour questionnaire (mean (SD)) |  |  |  |  |
| Extraversion | 5.14 (0.58) | 5.04 (0.70) | 5.14 (0.58) | 4.03 (0.43) |
| Negative affectivity | 3.37 (0.67) | 3.23 (0.58) | 3.37 (0.67) | 4.34 (0.60) |
| Effortful control | 4.72 (0.80) | 4.74 (0.58) | 4.75 (0.80) | 4.78 (0.83) |

*Note:* Mann-Whitney U Test was used to test median and Interquartile range (IQR) for Infant bonding (MIBS) by groups; t-test was used to explore mean and SD differences between groups

*p≤0.05, **p≤0.01, ***p≤0.001.

**Table S3. Spearman correlation between maternal sensitivity (Care index subscales), ED psychopathology (EDE-Q), depressive symptoms (BDI), Anxiety symptoms (STAI) in ED group**

|  | **Restraint subscale**  **N=21** | **Eating concern subscale**  **N=21** | **Weight concern**  **N=20** | **Shape concern**  **N=20** | **STAI-STATE**  **N=29** | **STAI-TRAIT**  **N=28** | **BDI**  **N=27** |
| --- | --- | --- | --- | --- | --- | --- | --- |
| **Mother sensitive** | Rs=-0.43 | Rs=-0.29 | Rs=-0.18 | Rs=-0.16 | Rs=-0.22 | Rs=-0.32 | Rs=0.24 |
| **Mother controlling** | Rs = 0.19 | Rs = 0.19 | Rs=0.12 | Rs=0.19 | Rs=0.03 | Rs=0.25 | Rs=-0.20 |
| **Mother unresponsive** | Rs=-0.22 | Rs=-0.33 | Rs=-0.17 | Rs=-0.05 | Rs=0.10 | Rs=0.09 | Rs=-0.24 |
| **Infant cooperative** | Rs = -0.07 | Rs =-0.11 | Rs =-0.09 | Rs =-0.21 | Rs=-0.27 | Rs=-0.36 | Rs=0.03 |
| **Infant difficult** | Rs =0.60 | Rs =0.24 | Rs =0.40 | Rs =0.46 | Rs=0.29 | Rs=0.22 | Rs=-0.20 |
| **Infant compulsive** | Rs = 0.08 | Rs =0.08 | Rs =0.08 | Rs =0.05 | Rs=-0.32 | Rs=-0.13 | Rs=-0.12 |
| **Infant passive** | Rs = -0.53 | Rs =-0.25 | Rs =-0.47 | Rs =-0.39 | Rs=0.15 | Rs=0.13 | Rs=0.00 |

*p ≤ 0.05; **p ≤ 0.01; ***p ≤ 0.001.

**Table S4. Spearman correlation between infant temperament at 1 year and ED psychopathology (EDE-Q) at 6 months postnatally, depressive symptoms (BDI), Anxiety symptoms (STAI) within C-ED group**

| **Predictor** |  | **Negative affectivity** | **Extraversion** | **Effortful control** |  | **Negative affectivity** | **Extraversion** | **Effortful control** |
| --- | --- | --- | --- | --- | --- | --- | --- | --- |
|  | **N Tot** | **Year 1** | | | **N Tot** | **Year 2** | | |
| **Restrain subscale** | 7 | **Rs=0.54*** | Rs=-0.07 | Rs=-0.78 | 7 | **Rs=0.43*** | Rs=0.02 | Rs=0.34 |
| **Eating concern subscale** | 7 | Rs=0.32 | Rs=-0.04 | Rs=-0.63 | 7 | Rs=0.62 | Rs=-0.51 | Rs=0.34 |
| **Weight concern subscale** | 7 | **Rs=0.67**** | Rs=-0.11 | Rs=-0.87 | 7 | Rs=0.51 | Rs=0.21 | Rs=0.04 |
| **Shape concern subscale** | 7 | **Rs=0.43*** | Rs=-0.14 | Rs=-0.43 | 7 | Rs=0.41 | Rs=0.20 | Rs=0.25 |
| **Composite STAI-STATE** | 7 | Rs=0.31 | Rs=-0.56 | Rs=-0.36 | 7 | Rs=0.30 | Rs=0.60 | Rs=0.24 |
| **Composite STAI-TRAIT** | 7 | Rs=0.07 | Rs=-0.36 | Rs=-0.11 | 7 | Rs=0.08 | Rs=0.53 | Rs=-0.13 |
| **Composite BDI** | 7 | Rs=0.07 | Rs=-0.46 | Rs=-0.04 | 7 | Rs=-0.00 | Rs=0.49 | Rs=0.14 |
|  |  |  |  |  |  |  |  |  |

*p≤0.05, *p **p≤0.01, ***p≤0.001. Comparison of mothers with history of C-ED only and P-ED only represents additional post hoc testing; p-values presented are adjusted using Bonferroni correction to account for multiple comparisons

**Table S5. Spearman correlation between infant temperament at 1 year and ED psychopathology (EDE-Q) at 6 months postnatally, depressive symptoms (BDI), Anxiety symptoms (STAI) within P-ED group**

| **Predictor** |  | **Negative affectivity** | **Extraversion** | **Effortful control** |  | **Negative affectivity** | **Extraversion** | **Effortful control** |
| --- | --- | --- | --- | --- | --- | --- | --- | --- |
|  | **N Tot** | **Year 1** | | | **N Tot** | **Year 2** | | |
| **Restrain subscale** | 13 | **Rs=0.72**** | Rs=-0.26 | Rs=-0.16 | 13 | **Rs=0.58*** | Rs=-0.53 | **Rs=0.62*** |
| **Eating concern subscale** | 13 | **Rs=0.80***** | Rs=-0.51 | Rs=-0.20 | 13 | Rs=0.21 | Rs=-0.51 | Rs=0.50 |
| **Weight concern subscale** | 13 | **Rs=0.66**** | Rs=-0.42 | Rs=-0.29 | 13 | Rs=0.14 | Rs=0.48 | Rs=0.47 |
| **Shape concern subscale** | 13 | **Rs=0.72**** | Rs=-0.53 | Rs=-0.27 | 13 | Rs=0.21 | Rs=0.48 | Rs=0.44 |
| **Composite STAI-STATE** | 13 | Rs**=**0.56 | Rs=-0.02 | Rs=-0.26 | 13 | Rs=-0.42 | Rs=-0.25 | Rs=0.27 |
| **Composite STAI-TRAIT** | 13 | **Rs=0.60**** | Rs=-0.21 | Rs=-0.34 | 13 | Rs=-0.46 | Rs=0.38 | Rs=0.16 |
| **Composite BDI** | 13 | Rs=0.56 | Rs=-0.29 | Rs=-0.11 | 13 | Rs=-0.46 | Rs=0.20 | Rs=0.31 |

*p≤0.05, **p≤0.01, ***p≤0.001. Comparison of mothers with history of C-ED only and P-ED only represents additional post hoc testing; p-values presented are adjusted using Bonferroni correction to account for multiple comparisons
